# *Oryza sativa COI* Homologues Restore Jasmonate Signal Transduction in *Arabidopsis coi1-1* Mutants

**DOI:** 10.1371/journal.pone.0052802

**Published:** 2013-01-08

**Authors:** Han Yong Lee, Ju-Seok Seo, Jang Hee Cho, Harin Jung, Ju-Kon Kim, Jong Seob Lee, Sangkee Rhee, Yang Do Choi

**Affiliations:** 1 Department of Agricultural Biotechnology, Seoul National University, Seoul, Korea; 2 School of Biotechnology and Environmental Engineering, Myongji University, Yongin, Korea; 3 School of Biological Sciences, Seoul National University, Seoul, Korea; University of Iowa, United States of America

## Abstract

*CORONATINE INSENSITIVE 1* (*COI1*) encodes an E3 ubiquitin ligase complex component that interacts with JAZ proteins and targets them for degradation in response to JA signaling. The Arabidopsis genome has a single copy of *COI1*, but the *Oryza sativa* genome has three closely related *COI* homologs. To examine the functions of the three OsCOIs, we used yeast two-hybrid assays to examine their interactions with JAZ proteins and found that OsCOIs interacted with OsJAZs and with JAZs, in a coronatine dependent manner. We also tested whether *OsCOI1a* and *OsCOI1b* could complement Arabidopsis *coi1-1* mutants and found that overexpression of either gene in the *coi1-1* mutant resulted in restoration of JA signal transduction and production of seeds, indicating successful complementation. Although OsCOI2 interacted with a few OsJAZs, we were not able to successfully complement the *coi1-1* mutant with OsCOI2. Molecular modeling revealed that the three OsCOIs adopt 3D structures similar to COI1. Structural differences resulting from amino acid variations, especially among amino acid residues involved in the interaction with coronatine and JAZ proteins, were tested by mutation analysis. When His-391 in OsCOI2 was substituted with Tyr-391, OsCOI2 interacted with a wider range of JAZ proteins, including OsJAZ1, 2, 5∼9 and 11, and complemented *coi1-1* mutants at a higher frequency than the other *OsCOI*s and *COI1*. These results indicate that the three *OsCOI*s are orthologues of *COI1* and play key roles in JA signaling.

## Introduction

Jasmonates (JA) are important hormones in the regulation of plant growth, development, defense and stress responses [1], [2], [3], [4], [5]. JA also plays a critical role in male fertility. Mutants defective in JA biosynthesis, such as *fad* [6], *opr3* [7], *dde1* [8], *dad1* [9] and *aos* [10], showed phenotypes of reduced anther filament elongation and delayed- or non-dehiscence. Application of exogenous JA to *opr3* mutant plants restored fertility but the JA precursor 12-oxo-phytodienoic acid (OPDA) did not restore fertility; thus, JA signaling leads to the elongation of anther filaments and production of fertile pollen [7]. Mutants affecting genes in the JA signaling pathway, such as *coi1* [11], *myb21* [12], *myb24* [13] and *myb26* [14], also showed inhibited filament elongation, reduced pollen development and lack of dehiscence.

JA signaling is mediated by CORONATINE INSENSITIVE 1 (COI1), which was identified based on its insensitivity to the phytotoxin JA analog coronatine [11]. COI1 acts as a JA receptor to initiate JA signaling [15], [16]. It is an F-box protein component of the Skp1-Cul-F-box protein (SCF) ubiquitin E3 ligase complex. COI1 interacts with JASMONATE ZIM DOMAIN (JAZ) family proteins in a JA-dependent manner, as an integral part of JA-mediated signal transduction [17]. JAZ proteins are recruited to the SCF^COI1^ complex and degraded through the 26S proteasome to promote the expression of JA responsive genes [15], [18], [19]. The Arabidopsis genome encodes one COI and 12 members of the JAZ family. JAZ 1, 3, 9 and 10 interact with COI in a jasmonoyl isoleucine (JA-Ile) or coronatine dependent manner [15], [18].

The *coi1-1* mutant has a point mutation of G to A at position +1401 [17]. This mutant exhibits a male sterile phenotype including inhibited filament elongation, and non-dehiscence [11], [17], [20]. It does not respond to JA-Ile or coronatine and is impaired in JA responses because JAZ proteins are not degraded in the presence of JA-Ile or coronatine [16].

There are three closely related *COI1* homologs in rice: *OsCOI1a* (Os01g0853400; AK121543), *OsCOI1b* (Os05g0449500; AK101514), and *OsCOI2* (Os03g0265500; AK100694). OsCOI1a was reported to form an SCF complex and regulate *OsbHLH148* expression in response to coronatine [21]. OsbHLH148-OsJAZ1-OsCOI1 constitutes a JA signaling module in *Oryza sativa* and OsJAZ1 is degraded by the SCF^OsCOI1^ complex-mediated 26S proteasome. *OsCOI1a* and *OsCOI1b* were also shown to be necessary for JA responses by RNA interference (RNAi) [22], [23].

In this study, we demonstrate the functional features of the three *COI* homologs of *Oryza sativa*. OsCOI1a, OsCOI1b and OsCOI2(H391Y) interacted with OsJAZ proteins and complemented the Arabidopsis *coi1-1* mutant. Complemented *coi1-1* plants recovered JA signal transduction and seed production capacity. Our results show that the three *OsCOIs* play a critical role in JA signaling in rice.

## Results

### The Rice (Oryza sativa L.) Genome Contains three COI1 Homologs

**Figure 1:**
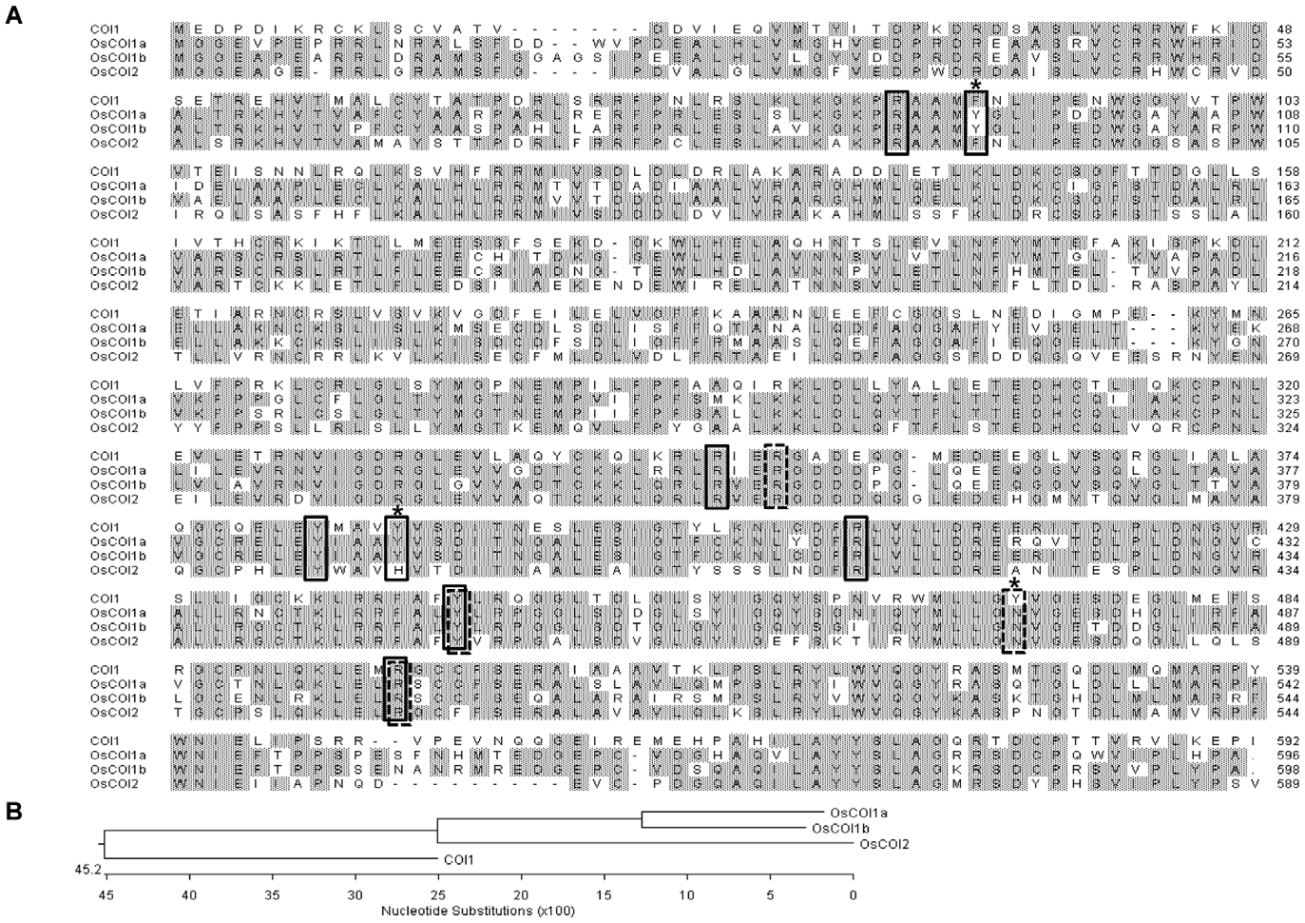
Amino acid sequence alignment and phylogenetic tree of COI1 and OsCOIs. A, Deduced amino acid sequences of COI1 and OsCOIs were aligned using the ClustalW program. Gray shading indicates identical residues. Approximately 55% identity was shown between COI and OsCOIs. Solid boxes indicate residues involved in the COI-coronatine complex. Dashed boxes indicate residues involved in the COI-JAZ interaction. Asterisks indicate amino acid residues involved in the COI-coronatine complex or COI-JAZ interaction, which shows the difference between coronatine and JAZ interactions. B, The phylogenetic tree of COI1 and OsCOIs. These results were drawn with DNAstar software.

There are three closely related *COI1* homologs in rice; *OsCOI1a* (Os01g0853400; AK121543), *OsCOI1b* (Os05g0449500; AK101514), and *OsCOI2* (Os03g0265500; AK100694) (Fig. 1). By contrast, Arabidopsis has only a single *COI* gene, *COI1*. COI1 and OsCOIs share approximately 55% amino acid sequence identity. The OsCOIs showed higher amino acid sequence identity to each other than to COI1. The identity between OsCOI1a and OsCOI1b was approximately 82% but the identity between OsCOI1a or OsCOI1b and OsCOI2 was approximately 63% (Fig. 1B and Table S1). Amino acid sequence similarities, including related amino acids, between COI1 and OsCOIs were approximately 75%. The similarity between OsCOI1a and OsCOI1b was approximately 89% but the similarity between OsCOI1a or OsCOI1b and OsCOI2 was approximately 75% (data not shown).

### All 3 OsCOIs are Expressed

**Figure 2:**
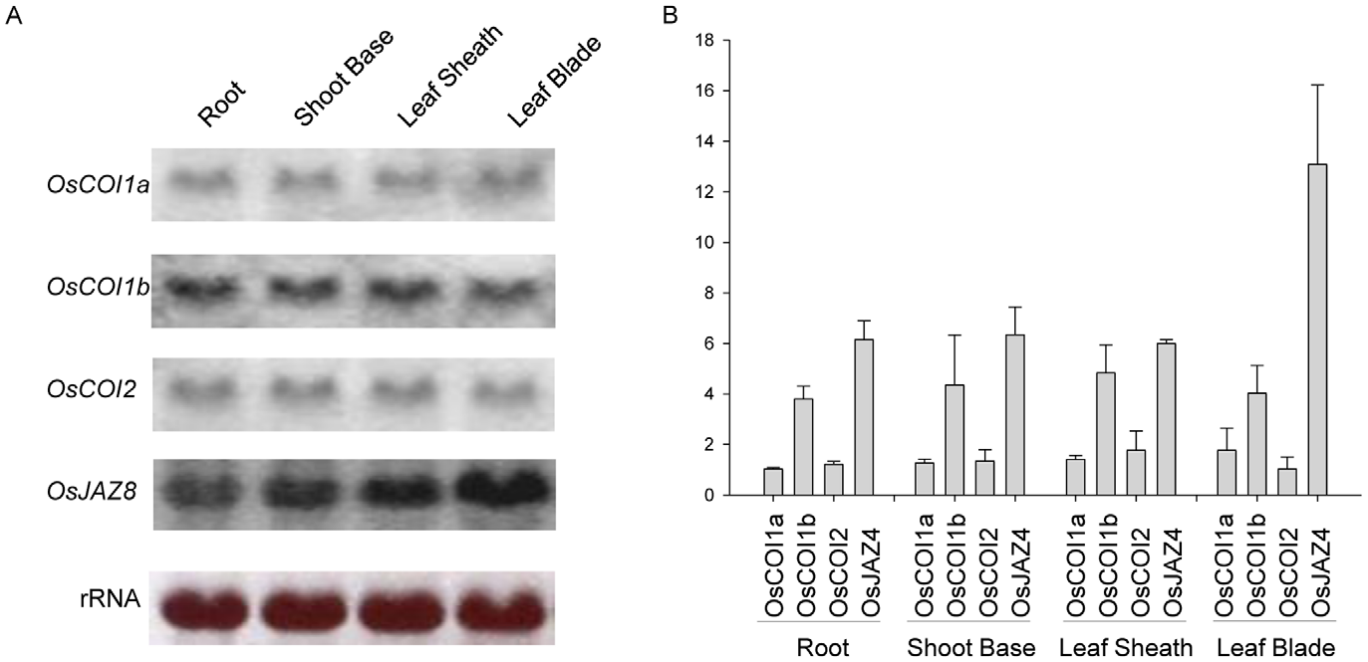
Expression levels of *OsCOIs.* Relative expression levels of *OsCOI1a*, *OsCOI1b* and *OsCOI2*, as shown by qRT-PCR. Expression level of *OsCOI1a* was set to 1 arbitrarily and the relative expression levels of *OsCOI1b* and *OsCOI2* are shown. Data represent mean values of three measurements and error bars represent standard deviation. Total RNA was isolated from 2-week-old seedlings and equal amounts of RNA were used for qRT-PCR analysis.

To test the basal expression of the three *OsCOIs*, we carried out Northern blot analysis and qRT-PCR with total RNA isolated from various tissues of 2-week-old seedlings of wild type rice. All three *OsCOIs* were expressed but the expression level of *OsCOI1b* was the higher than the others and those of *OsCOI1a* and *OsCOI2* were similar to each other (Fig. 2). Expression levels of those genes were similar in various tissues such as root, shoot base, leaf sheath and leaf blade.

### Molecular Modeling of OsCOI Structure

Based on the relatively high sequence identity of OsCOIs to COI1, we carried out molecular modeling study of the three OsCOIs with SWISS-MODELER using the structure of COI1 in complex with coronatine and the peptide for a JAZ degron (PDB ID 3OGM) [24], as a template (Fig. S1). Overall, the three OsCOIs exhibited structures similar to that of COI1, each with a root mean square deviation of 0.34, 0.33 and 0.34 Å, respectively. In particular, the OsCOIs also contain the putative binding site for coronatine and JAZ, but sequence variations in OsCOIs presented minor differences in those sites compared to COI1.

For the coronatine-binding site in COI1, the cyclopentanone ring in coronatine is bound in the pocket enclosed by Phe-89, Tyr-386, and Tyr-444, with stacking interactions between Phe-89 and Tyr-444, and its keto group is within hydrogen bonding distance of Tyr-444 and Arg-496 (Fig. S2). The remaining amide and terminal carboxyl moiety is embedded into the concavity formed by Arg-85, Arg-348, and Arg-409 with additional possible hydrogen bonds of less than 3.2 Å. These structural features were well conserved in the OsCOIs, as indicated by the sequence alignment (Fig. 1A and Fig. S2).

**Figure 3:**
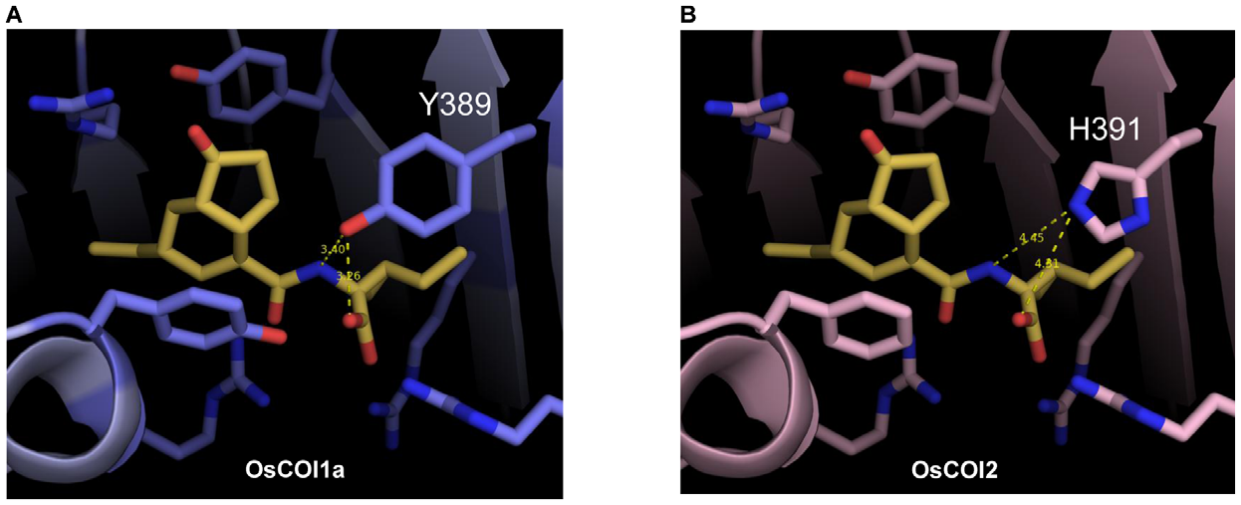
Molecular modeling of the OsCOI-coronatine complex. A, OsCOI1a-coronatine complex. B, OsCOI2-coronatine complex. COI1, OsCOI1a, and OsCOI1b have Tyr-386, Tyr-389, or Tyr-391 residues, respectively, but OsCOI2 has a His-391 residue at the interaction point. Hydrogen bonds are shown as yellow dotted lines. COI1, OsCOI1a and OsCOI1b form a 3.4 Å hydrogen bond but OsCOI2 forms a 4.45 Å hydrogen bond.

Noticeable differences include, however, the two residues equivalent to the Phe-89 and Tyr-386 in COI1. In OsCOI1a and 1b, there was only one variation in the coronatine-binding site residues, compared with COI1 (Fig. S2A and S2B), the tyrosine residue (Tyr-94 and Tyr-96, respectively) structurally equivalent to Phe-89 in COI1. This replacement is unlikely to affect functions of these two OsCOIs; the aromatic ring in the tyrosine residue could still maintain stacking interactions with the cyclopentanone ring of coronatine, as described for COI1. This variation may produce additional hydrogen binding with the carbonyl oxygen between the cyclopentanone ring and terminal carboxyl moiety of coronatine. Unlike OsCOI1a and OsCOI1b, OsCOI2 contains one replacement of His-391 at the position corresponding to Tyr-386 in COI1 (Fig. 3). In particular, Tyr-386 in COI1 contributes to the stabilization of the binding of coronatine by forming a hydrogen bond to the amine group. However, in OsCOI2, His-391 is distant from coronatine, about 4.5 Å, likely precluding this stabilization interaction.

For the binding site for a JAZ degron peptide, the OsCOIs have essentially identical structural environments, except for one change corresponding to Tyr-472 in COI1 (Fig. S3D). In COI1, Tyr-472 is within the distance for hydrogen bonding (3.1 Å) with the backbone oxygen of Leu-201 in JAZ1, but that possible interaction is unlikely in these three OsCOIs, which have a relatively small-chain asparagine residue in that position. The current model indicates that Asn-475 in OsCOI1a, Asn-477 in OsCOI1b, and Asn-477 in OsCOI2 are distant from the backbone oxygen of Leu-201 in JAZ1 (Fig. S3A, S3B and S3C).

### OsCOIs Interact with OsJAZs and JAZs

To determine whether OsCOIs interact with JAZs in JA signal transduction, yeast two hybrid assays were performed. We found that the three OsCOIs interact with most of the OsJAZs and JAZs in a coronatine dependent manner (Figs. S4 and S5 and summarized in Table 1 and Table S2). OsCOI1b interacts with the widest range of OsJAZs and JAZs in a coronatine-dependent manner. OsCOI2 interacts with only a few of the OsJAZs but with none of the JAZs.

**Table 1:** Summary of the Y2H assay of OsJAZs.

	1	2	3	4	5	6	7	8	9	10	11	12
OsCOI1a	+++^1^	+	+	−	++	++	−	++	++	−	++	−
OsCOI1a(N475)^2^	+++	+	−	−	++	+	−	+++	+++	++	+	−
OsCOI1b	+++	+++	+++	++	+++	+++	+++	+++	+++	+++	−	−
OsCOI2	+	++	−	−	−	−	−	−	−	−	+++	−
OsCOI2(H391Y)^3^	+++	+++	−	−	+	++	+	+++	+++	−	+++	−
OsCOI2(F91Y)^4^	+	+++	−	−	−	+++	++	−	+	−	+++	−
OsCOI2(N477Y)^5^	−	++	−	−	−	+	+	−	−	−	++	−
OsCOI2(F91Y, H391Y, N477Y)^6^	+++	++	−	−	+++	+++	+	+++	+++	+	++	−
COI1^7^	++	−	−	−	++	++	−	+++	++	++	−	−

1 The strength of each interaction was rated as strong (+++), medium (++), weak (+) or undetectable (−), as shown in Figure S4.

2 OsCOI1a(N475Y) is a point mutant in which asparagine at 475 has been changed to tyrosine.

3 OsCOI2(H391Y) is a point mutant in which histidine at 391 has been changed to tyrosine.

4 OsCOI2(F91Y) is a point mutant in which phenylalanine at 91 has been changed to tyrosine.

5 OsCOI2(N477Y) is a point mutant in which asparagine at 477 has been changed to tyrosine.

6 OsCOI2(F91Y, H391Y, N477Y) is a point mutant in which each amino acid at there position has been changed to tyrosine.

7 [21].

To evaluate the functional consequences of the minor sequence variations in OsCOIs binding sites, we also examined wild type and various mutant OsCOIs. OsCOIs mutated according to the molecular models were tested in parallel by yeast two hybrid assays. When His-391 in OsCOI2 was substituted with Tyr-391 as in OsCOI2(H391Y), it interacted with a wider range of OsJAZs, including OsJAZ1, 2, 5∼9 and 11. It also interacted with JAZ1∼4, 9, 11 and 12 in a coronatine-dependent manner. None of OsJAZs or JAZs interacted with the OsCOIs in Y2H assay in the presence of JA or MeJA (data not shown).

Mutation of Phe-91 of OsCOI2 to Tyr-91, to generate OsCOI2(F91Y), made it interact with OsJAZ6 and 7 in addition to its other JAZ interactions. OsCOI2(F91Y) also interacted with JAZ3, 4 and 9. Structural modification widened the OsCOI2 interaction spectrum and enhanced its interactions with JAZs. However, OsCOI2(N477Y) and OsCOI1a(N475Y) did not show much difference in interaction with JAZs, if any. The triple mutant OsCOI2(F91Y, H391Y, N477Y) was similar to OsCOI2(H391Y), with a slight enhancement of JAZ interactions.

### Overexpression of OsCOI1a and OsCOI1b Restores Fertility of Arabidopsis *coi1-1* Mutants

**Figure 4:**
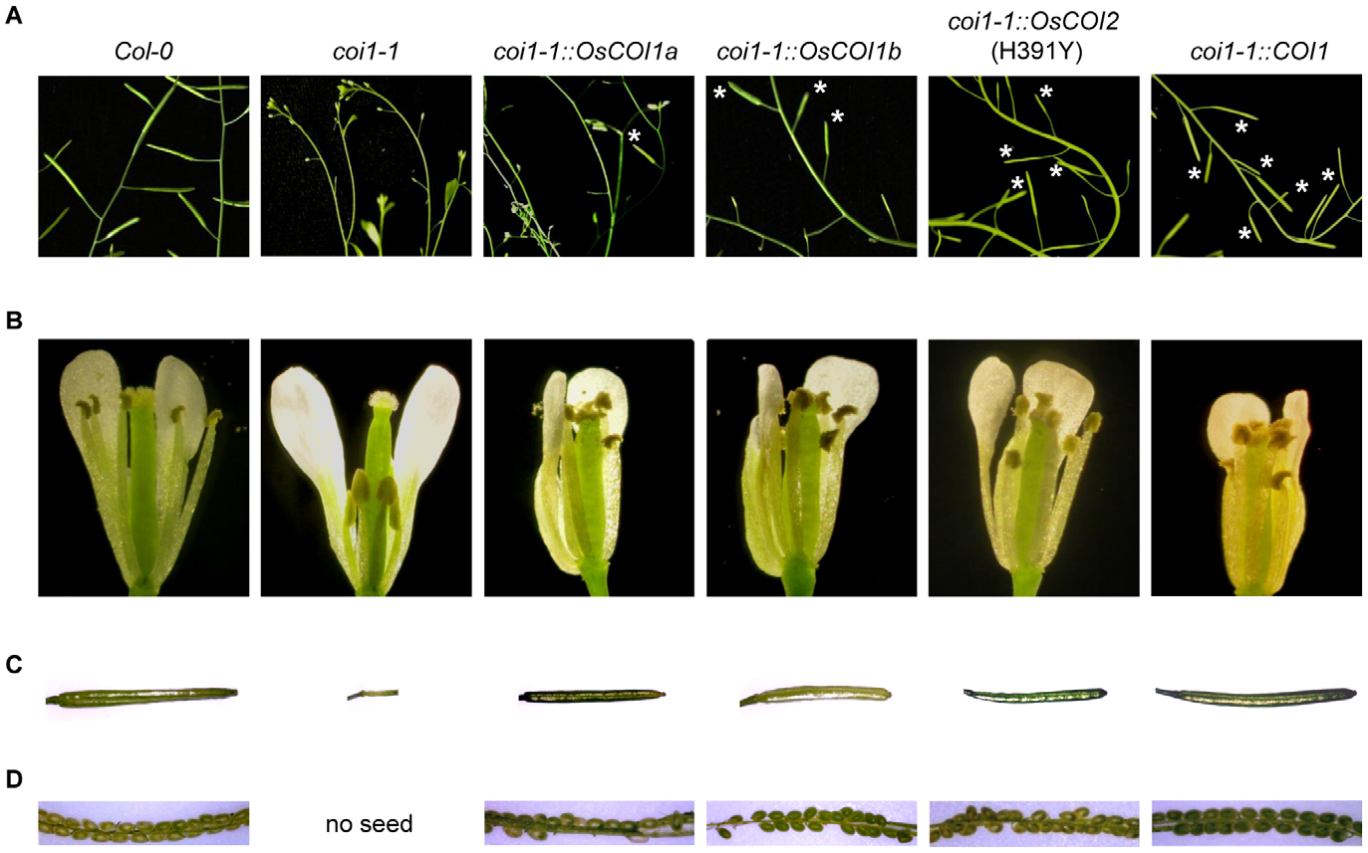
Morphological phenotype of complemented *coi1-1*. The *coi1-1* mutant was complemented with *OsCOI1a* (#1134), *OsCOI1b* (#2124), *OsCOI2*(H391Y) (#H3159) or *COI1* (#A0088), respectively, at the T2 generation. A, Siliques of 6-week-old plants grown in soil. The asterisks indicate developing siliques. B, Flowers of 6-week-old plants grown in soil. C, Fully developed siliques. D, Developing seeds in the silique.

To test whether the OsCOIs can function in JA signal transduction, we tested whether they could complement the Arabidopsis *coi1-1* mutant. Genetic complementation was accessed by restoration of fertility. Each of the *OsCOIs*, including the *OsCOI2*(H391Y) mutant, was combined with the 35S promoter and transformed into *coi1-1* heterozygous F1 plants. *coi1-1* homozygous mutant segregants were identified in the progeny by PCR. Overexpression of *OsCOI1a* and *OsCOI1b* complemented *coi1-1* homozygous mutant segregants, which were now fertile and produced seeds (Fig. 4). The size and shape of flowers from complemented plants were similar to those of wild type. We could not find any pollen in *coi1-1* mutant flowers, nor normal siliques. By contrast, homozygous *coi1-1* Arabidopsis that were complemented by *OsCOI1a* or *OsCOI1b* made viable pollen and normal siliques containing seeds. However, complementation by OsCOIs was less efficient than by COI1 because not all siliques were fully developed and produced seeds as shown in Figure 4.

Expression levels of the transgene in the various transformant lines was analyzed by Northern blot (Fig. S6). The degree of fertility complementation and JA response increased with increasing levels of transgene expression. The number of copies of each transgene was also assessed by genomic Southern Blot analysis (Fig. S7). Transformants containing a single copy of the transgene and maintaining a relatively high level of expression were selected for further studies.

### The *OsCOI2*(H391Y) Mutant Complemented *coi1-1* at an Increased Frequency

In contrast to *OsCOI1a* and*OsCOI1b*, we could not obtain any *coi1-1* plants complemented by *OsCOI2* (Table 2). None of the 38 *coi1-1* homozygous segregants transformed with *OsCOI2* made productive siliques or seeds. They also did not show any response to MeJA. By contrast, constructs overexpressing the mutant *OsCOI2*(H391Y), did complement *coi1-1.* Overexpression of *OsCOI2*(H391Y) in *coi1-1* plants resulted in plants that make seeds, as was observed for *OsCOI1a* and *OsCOI1b* (Fig. 4).

**Table 2:** Segregation of *coi* genotypes in transformants and their complementation frequency.

Transgene	Genotype of transformed Arabidopsis^1^				Complemented *coi1-1/coi1-1*	
	*COI1/COI1* (%)	*COI1/coi1-1* (%)	*coi1-1/coi1-1* (%)	Total (100%)	No of lines^2^	Frequency^3^
*OsCOI1a*	43 (25.1%)	84 (49.1%)	44 (25.7%)	171 (100%)	5/44	11.4%
*OsCOI1b*	25 (24.5%)	50 (49.0%)	27 (26.5%)	102 (100%)	6/27	22.2%
*OsCOI2*	39 (25.2%)	78 (50.3%)	38 (24.5%)	155 (100%)	0/38	0
*OsCOI2*(H391Y)	25 (28.4%)	39 (44.3%)	24 (27.3%)	88 (100%)	8/10^4^	80.0%
*COI*	26 (29.5%)	42 (47.8%)	20 (22.7%)	88 (100%)	10/20	50.0%

1 Number of independently transformed Arabidopsis lines selected on BASTA containing media.

2 Number of complemented lines out of transformed *coi1-1/coi1-1* segregants.

3 Per cent of complemented lines among transformed *coi1-1/coi1-1* segregants.

4 Ten out of 24 *OsCOI2*(H391Y) lines were selected randomly and analyzed for complementation.

The mutant *OsCOI2*(H391Y), moreover, complemented *coi1-1* at a higher frequency than *COI1* and other *OsCOIs*. Twenty-four lines of segregated homozygous *coi1-1* mutants were selected from 88 transformed lines. Only ten out of twenty-four transformed segregants were selected randomly for further analysis. Eight out of ten homozygous transformant lines made seeds, a complementation frequency of 80% (Table 2). This frequency was higher than the frequencies observed for *OsCOI1a* and *OsCOI1b* of 11% and 22%, respectively, and even higher than that for COI1, which was 50%.

### Overexpression of *OsCOIs* Restores the JA Response

**Figure 5:**
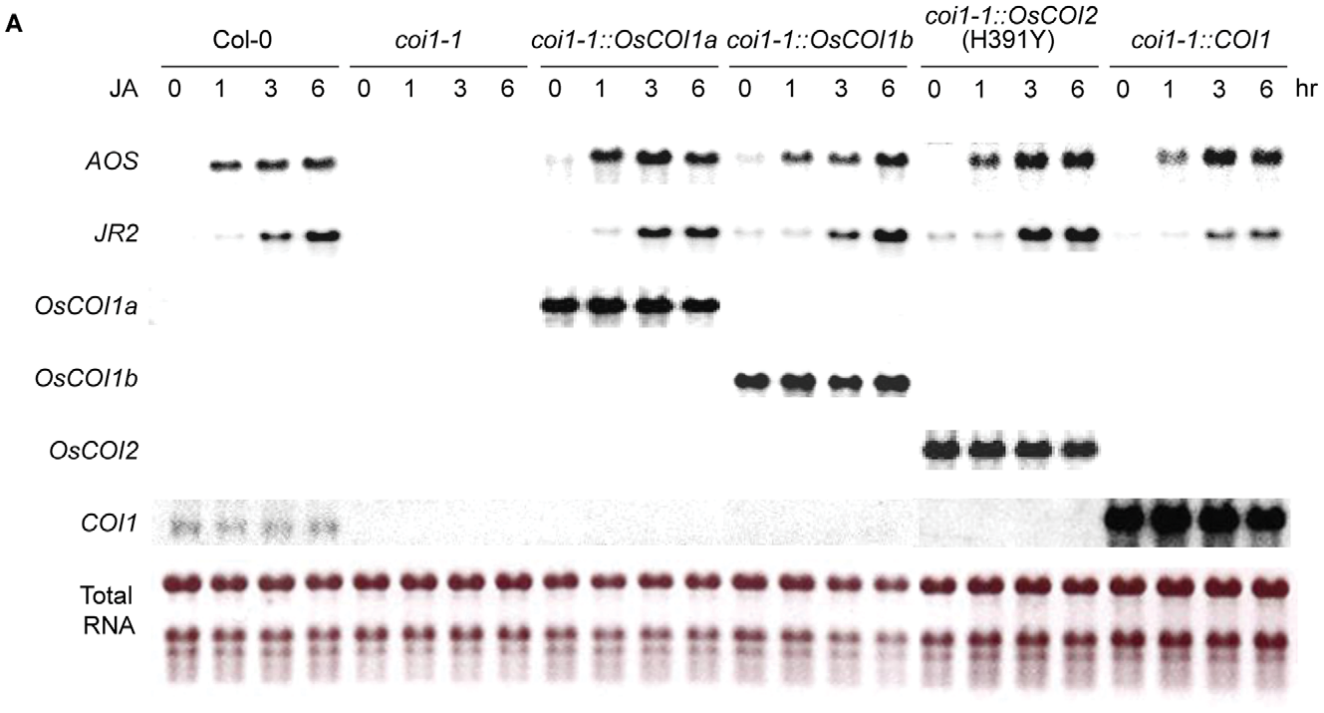
Inducible expression of JA responsive genes by complementation. Response of *AOS* and *JR2* is shown in the *coi1-1* mutant transformed with *OsCOI1a* (#1134), *OsCOI1b* (#2124), *OsCOI2*(H391Y) (#H3159) or *COI1* (#A0088), respectively, at T2 generation. Plants were sprayed with 50 µM MeJA and total RNA was isolated at the indicated times after MeJA treatment and analyzed by Northern blot. rRNA was visualized by ethidium bromide staining to show an equal loading. No complemented plants were obtained using *OsCOI2* in this experiment.

To understand the molecular mechanism of fertility restoration, we tested whether overexpression of OsCOIs restored the JA response in *coi1-1*. To test the JA response, 5-week-old complemented Arabidopsis at the T3 generation were treated with 50 µM MeJA. The MeJA response marker genes *AOS* and *JR2* were not expressed in *coi1-1* mutants, but were induced at 3 hr after MeJA treatment in complemented *coi1-1* rosette leaves and in wild type (Fig. 5). MeJA responsive gene expression was observed only after the expression of transgenes overexpressing *OsCOI1a, OsCOI1b, OsCOI2*(H391Y) or *COI1*. The basal level of endogenous COI1 expression was relatively much lower than that in transformants. In *coi1-1* mutants transformed with*OsCOI2*, *AOS* and *JR2* were not induced by MeJA, consistent with the failure of *OsCOI2* to complement the *coi1-1* male sterile phenotype (Fig. S6B). Eight out of ten lines that were complemented with *OsCOI2*(H391Y), however, responded to MeJA (Fig. 5). The MeJA responsiveness and fertility were also restored in other complemented lines (Fig. S6).

**Figure 6:**
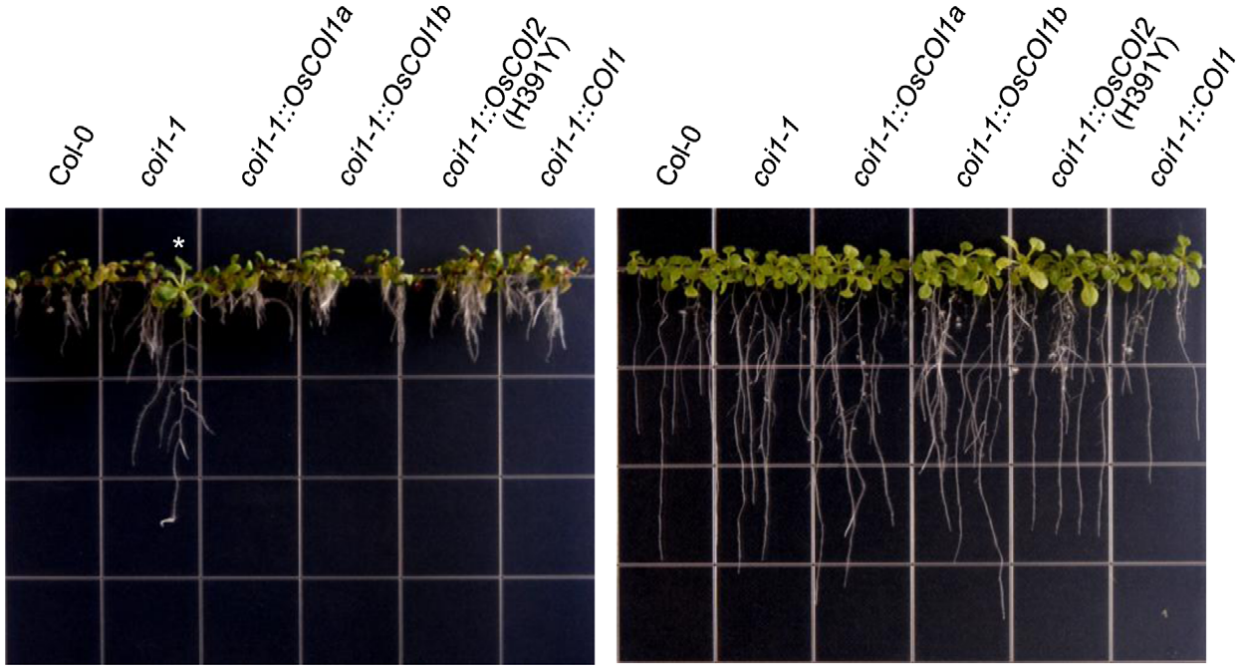
Restoration of root growth inhibition phenotype by complementation. Col-0, *coi1-1* heterozygote, and complemented T3 homozygous lines were grown vertically on MS media containing 50 µM MeJA (left) or without MeJA (right). The asterisk indicates *coi1-1* homozygote which was tested by PCR and *Xcm*I enzyme digestion.

Overexpression of OsCOIs also restored the root growth inhibition phenotype as shown in Figure 6. When plants were grown on MS medium containing 50 µM MeJA, all complemented lines including heterozygous *coi1-1* showed root growth inhibition in response to JA. Homozygous *coi1-1* did not show root growth inhibition.

## Discussion

There are three closely related *COI1* homologs in rice. In this study, we demonstrate the function of those rice *COI* homologs in JA signal transduction by complementation of the Arabidopsis *coi1-1* mutant, which is impaired in JA responses including fertility. As knockout mutants of *OsCOIs* were not available, overexpression of OsCOIs driven by the 35S promoter was accomplished by stable transformation of the *coi1-1* mutant, in which JA signal transduction and fertility was restored. These results demonstrate that these OsCOI homologs are orthologues of *COI1*.

OsCOIs were shown by molecular modeling to form 3D structures similar to COI1 [24]. These structural features suggest that OsCOIs could function in the JA signaling pathway in rice as COI1 functions in Arabidopsis. Even though the overall 3D structures of the OsCOIs were quite similar to COI1, there were some variations in the amino acids that interact with coronatine or JA-Ile. Structural variations of these OsCOIs were tested by mutant analysis in yeast two hybrid assays and by transformation into Arabidopsis.

According to yeast two hybrid assays, the OsCOIs showed different specificities of interaction with members of the OsJAZ family (Fig. S4 and Table 1). OsCOI1b interacted with the widest range of OsJAZs but OsCOI2 interacted with a limited set of OsJAZs. These results suggest that *OsCOI1b* may play the major role among the three *OsCOIs* in rice. It is still possible, however, that Arabidopsis background affects the complementation frequency, especially for OsCOI2, which did not show complementation in this experiment. Also lower efficiency of complementation by individual OsCOIs might be attributed to different specificity of each OsCOIs for JAZ and thus target genes. These results suggest that the interaction specificities between OsCOIs and OsJAZs may determine cellular response to different signal pathways. This may explain the presence of 3 homologues in rice differently from Arabidopsis in which a single copy of COI1 is present. It is consistent with general features in eukaryotic genome in which duplication and diversification of genome is driven with evolution.

It is noteworthy to mention that the specificity and function of OsCOIs could be modulated by mutation. For example, OsCOI2(H391Y) complemented with higher frequency than the other OsCOIs, including wild type. It is also interesting that mutation of an amino acid in the binding pocket for coronatine affects the specificity of the JAZ interaction. It is possible for OsCOI2 to interact with different jasmonates, such as JA derivatives or analogues with bulkier functional group. It was shown by Y2H assay that the mutation resulted in wider interaction spectrum and stronger interaction with JAZs. Even though complementation frequency of was higher, complementation efficiency of mutant *OsCOI2*(H391Y) were lower than *COI1,* suggesting the importance of interface and its consequence for interacting with JAZs. Interaction specificity between COIs and JAZs could determine the efficiency of complementation.

The sequences of COI1 and OsCOIs that interact with the Jas/ZIM domains are very similar between rice and Arabidopsis and the Jas/ZIM domain sequences of JAZs and OsJAZs are also very similar between rice and Arabidopsis [21]. However, here we uncovered key amino acid sequence variations that conditioned differences in binding specificity. For example, OsCOI1b interacted with the widest range of OsJAZs even though it contains Asn-477, which is different from Tyr-472 in COI1 (Fig. 1, S3 and Table 1). When Asn-477 was mutated to Tyr-475 in OsCOI1a(N475Y) and Tyr-477 in OsCOI2(N477Y), the effect was less pronounced (Figs. S4, Fig. S5). These results suggest that Asn-475 of OsCOI1a and Asn-477 of OsCOI2 are involved in its interaction with OsJAZs but their contribution may be less important than that of other amino acids.

In conclusion, functional features of three OsCOIs were demonstrated in this study. JA signal transduction mediates diverse cellular responses and COIs are critical components in the response pathways. Manipulation of COI structure by mutation may contribute to enhanced stress resistance and grain yield especially in crop plants including rice.

## Materials and Methods

### Plant Materials and Growth Conditions

*OsCOI1a, OsCOI1b* and *OsCOI2* were obtained from the Rice Genome Resource Center, Japan. *Arabidopsis thaliana* ecotype Columbia (Col-0) was used as the wild type for all experiments. Heterozygous mutant *coi1-1* was kindly provided by J. Turner (University of East Anglia, Norwich, UK) [20]. Surface sterilized seeds were sown on MS medium containing 1% sucrose, 2.34 mM MES (pH5.7) and 0.7% agar, and chilled at 4°C for 3 days. Seeds were grown under 16 h day and 8 h night cycles at 22°C in a growth chamber. The soil-grown plants were placed in the same photoperiod and temperature.

For gene induction analyses, plants were sprayed with 50 µM MeJA (Sigma) in 1% ethanol, and were harvested after the indicated time.

### Molecular Modeling

Structures of the OsCOIs were modeled using SWISS-MODELER [25], with that of COI1 as a template [24], and were presented using PyMOL [26].

### Yeast two Hybrid Assays

The coding sequences (CDS) of *OsJAZ* genes were amplified by RT-PCR from 14-day-old seedlings of wild type *Oryza sativa* cultivar Nipponbare. The CDS of *JAZ* genes were amplified by RT-PCR from 14-day-old seedling of *Arabidopsis thaliana* plants, ecotype Columbia. Primer pairs for each gene are listed in Table S3. *OsJAZ*s and *JAZ*s were cloned into the Y2H prey vector pGADT7 (Clontech, http://www.clontech.com/). *OsCOI*s were cloned into the Y2H bait vector pGBKT7 (Clontech). Prey and bait constructs were co-transformed into *Saccharomyces cerevisiae* AH109. Co-transformed colonies were selected on synthetic dropout glucose medium (SD) without Leu and Trp (DDO). To confirm the OsCOIs-OsJAZs and OsCOIs-JAZs interactions, several co-transformed colonies (2 mm diameter) grown on DDO medium for 3 days were resuspended in 300 µl of autoclaved distilled H_2_O, and 30 µl of resuspended cells were dropped onto SD medium without Ade, His, Leu and Trp (QDO) in the presence of 30 µM JA, 30 µM MeJA or 100 µM coronatine (Sigma, http://www.sigmaaldrich.com/). The dropped cells were grown for 7 days in order to confirm the interaction.

### *coi1-1* Homozygote Selection

*coi1-1* homozygote plants were selected according to the protocol described by [20]. Genomic DNA was amplified by PCR (primers are listed in Table S3), and purified PCR products were digested with *Xcm*I, which could not recognize the mutant sequence.

### Southern and Northern Blot Analysis

Genomic DNA was prepared using the CTAB method [27]. For genomic Southern blot, restriction enzyme digested DNA was separated on 0.8% agarose gels, and transferred onto GeneScreen Plus hybridization transfer membranes (PerkinElmer, http://perkinelmer.com). cDNA probes were obtained by RT-PCR of RNA isolated from 2-week-old wild type rice leaves, labeled by random primer extension using [α-^32^P]dCTP (IZOTOP, http://www.izotop.hu).

Northern blot analysis was performed using total RNA extracted from frozen and ground samples using the phenol/SDS/LiCl method [28]. One µg of total RNA was separated on a 1.2% formaldehyde agarose gel and processed as for Southern blot analysis.

### qRT-PCR

cDNAs were obtained by RT-PCR of DNase treated (10 units for 1 hr at 25°C) RNA isolated from 2-week-old wild type rice leaves. The PCR was carried out in triplicates for 40 cycles of amplification (denature 15 seconds at 95°C, anneal 15 seconds at 50°C, extension 30 seconds at 72°C) on Rotor-Gene 2000 Real Time Amplification System (Corbett Research, http://www.corbettresearch.com) using the SYBR kit (JMC R&D, Seoul, Korea). *OsActin1* was employed as a reference in the assay for normalization.

### Accession Numbers

Rice Genome Initiative numbers for genes described in this article are as follows:

OsCOI1a (Os01g0853400; AK121543), OsCOI1b (Os05g0449500; AK101514), OsCOI2 (Os03g0265500; AK100694), OsJAZ1 (Os10g0392400; AK061602), OsJAZ2 (Os03g0180900; AK073589), OsJAZ3 (Os03g0180800; AK070649), OsJAZ4 (Os03g0181100; AK120087), OsJAZ5 (Os03g0402800; AK061842), OsJAZ6 (Os07g0615200; AK065604), OsJAZ7 (Os09g0439200; AK108738), OsJAZ8 (Os09g0401300; AK065170), OsJAZ9 (Os08g0428400; AK103459), OsJAZ10 (Os04g0653000; AK059441), OsJAZ11 (Os04g0395800; AK107750), OsJAZ12 (Os02g0732400; AK107003).

Arabidopsis Genome Initiative numbers for genes described in this article are as follows: COI1 (At2g39940), JAZ1 (At1g19180), JAZ2 (At1g74950), JAZ3 (At3g17860), JAZ4 (At1g48500), JAZ5 (At1g17380), JAZ6 (At1g72450), JAZ7 (At2g34600), JAZ8 (At1g30135), JAZ9 (At1g7070), JAZ10 (At5g13220), JAZ11 (At3g43440), JAZ12 (At5g20900), AOS (At5g42650), JR2 (At4g23600).

## Supporting Information

Figure S1 **Molecular modeling of OsCOI-coronatine complex and OsCOI-JAZ interaction.**
(PDF)

Figure S2 **Molecular modeling of COI-coronatine complex.**
(PDF)

Figure S3 **Molecular modeling of COI-JAZ interaction.**
(PDF)

Figure S4 **OsJAZs interact with OsCOIs in a coronatine-dependent manner in Y2H assays.**
(PDF)

Figure S5 **JAZs interact with OsCOIs in a coronatine-dependent manner in Y2H assays.**
(PDF)

Figure S6 **Expression of transgenes and restoration of JA response by complementation.**
(PDF)

Figure S7 **Genomic Southern blot analysis of complemented** ***coi1-1*** **mutants.**
(PDF)

Table S1 **The amino acid and nucleotide sequence identity of COI1 and OsCOIs.**
(PDF)

Table S2 **Summary of the Y2H assays with JAZs.**
(PDF)

Table S3 **Primers used in this study.**
(XLS)

## References

[1] Sasaki Y, Asamizu E, Shibata D, Nakamura Y, Kaneko T, et al (2001) Monitoring of methyl jasmonate-responsive genes in Arabidopsis by cDNA microarray:self-activation of jasmonic acid and biosynthesis and crosstalk with other phytohormone signaling pathways. DNA Res 8: 153–161. 11572481 10.1093/dnares/8.4.153

[2] Cheong J-J, Choi YD (2003) Methyl jasmonate as avital substance in plants. Trends Genet 19: 409–413. 12850447 10.1016/S0168-9525(03)00138-0

[3] Kim EH, Kim YS, Park S-H, Koo YJ, Choi YD, et al (2009) Methyl jasmonate reduces grain yield by mediating stress signals to alter spikelet development in rice. Plant Physiol 149: 1741–1760. 10.1104/pp.108.134684 PMC2663756 19211695

[4] Koo A, Howe GA (2009) The wound hormone jasmonate. Phytochemistry 70: 1571–1580. 19695649 10.1016/j.phytochem.2009.07.018 PMC2784233

[5] Acosta IF, Farmer EE (2010) Jasmonates. The Arabidopsis book. American Society of Plant Biologists, 1–13. 10.1199/tab.0129 PMC3244945 22303255

[6] McConn M, Browse J (1996) The critical requirement for linolenic acid is pollen development, not photosynthesis, in an Arabidopsis mutant. Plant Cell 8: 403–416. 12239389 10.1105/tpc.8.3.403 PMC161109

[7] Stintzi A, Browse J (2000) The Arabidopsis male-sterile mutant, opr3, lacks the 12-oxophytodienoic acid and reductase required for jasmonate synthesis. Proc Natl Acad Sci USA 97: 10625–10630. 10973494 10.1073/pnas.190264497 PMC27075

[8] Sanders PM, Lee PY, Biesgen C, Boone JD, Beals TP, et al (2000) The Arabidopsis delayed dehiscence1 gene encodes an enzyme in the jasmonic acid synthesis pathway. Plant Cell 12: 1041–1061. 10899973 10.1105/tpc.12.7.1041 PMC149048

[9] Ishiguro S, Kawai-Oda A, Ueda J, Nishida I, Okada K (2001) The DEFECTIVE IN ANTHER DEHISCENCE gene encodes a novel phospholipase A1 catalyzing the critical step of jasmonic acid biosynthesis, which synchronizes pollen maturation, anther dehiscence and flower opening in Arabidopsis. Plant Cell 13: 2191–2209. 11595796 10.1105/tpc.010192 PMC139153

[10] Park J-H, Halitschke R, Kim HB, Baldwin IT, Feldmann KA, et al (2002) A knock-out mutation in allene oxide synthase results in male sterility and defective wound signal transduction in Arabidopsis due to a block in jasmonic acid biosynthesis. Plant J 31: 1–12. 12100478 10.1046/j.1365-313x.2002.01328.x

[11] Feys B, Benedetti CE, Penfold CN, Turner JG (1994) Arabidopsis mutants selected for resistance to the phytotoxin coronatine are male sterile, insensitive to methyl jasmonate, and resistant to a bacterial pathogen. Plant Cell 6: 751–759. 12244256 10.1105/tpc.6.5.751 PMC160473

[12] Shin B, Choi G, Yi H, Yang S, Choi I, et al (2002) AtMYB21, a gene encoding a flower-specific transcription factor, is regulated by COP1. Plant J 30: 23–32. 11967090 10.1046/j.1365-313x.2002.01264.x

[13] Yang XY, Li JG, Pei M, Gu H, Chen ZL, et al (2007) Over-expression of a flower-specific transcription factor gene AtMYB24 causes aberrant anther development. Plant Cell Rep 26: 219–228. 16972096 10.1007/s00299-006-0229-z

[14] Steiner-Lange S, Unte US, Eckstein L, Yang C, Wilson ZA, et al (2003) Disruption of Arabidopsis thaliana MYB26 results in male sterility due to non-dehiscent anthers. Plant J 34: 519–528. 12753590 10.1046/j.1365-313x.2003.01745.x

[15] Thines B, Katsir L, Melotto M, Niu Y, Mandaokar A, et al (2007) JAZ repressor proteins are targets of SCFCOI1 complex during jasmonate signaling. Nature 448: 661–665. 17637677 10.1038/nature05960

[16] Katsir L, Chung HS, Koo AJ, Howe GA (2008) Jasmonate signaling: a conserved mechanism of hormone sensing. Curr Opin Plant Biol 11: 428–435. 18583180 10.1016/j.pbi.2008.05.004 PMC2560989

[17] Xie D, Feys BF, James S, Nieto-Rostro M, Turner JG (1998) COI1: an Arabidopsis gene required for jasmonate-regulated defense and fertility. Science 280: 1091–1094. 9582125 10.1126/science.280.5366.1091

[18] Chini A, Fonseca S, Fernandez G, Adie B, Chico JM, et al (2007) The JAZ family of repressor is the missing link in jasmonate signaling. Nature 448: 666–671. 17637675 10.1038/nature06006

[19] Chini A, Boter M, Solano R (2009) Plant oxylipins: COI/JAZs/MYC2 as the core jasmonic acid-signalling module. FEBS J 276: 4682–4692. 19663905 10.1111/j.1742-4658.2009.07194.x

[20] Xu L, Liu F, Lechner E, Genschik P, Crosby WL, et al (2002) The SCF^COI1^ ubiquitine-ligase complexes are required for jasmonate response in Arabidopsis. Plant Cell 14: 1919–1935. 12172031 10.1105/tpc.003368 PMC151474

[21] Seo J-S, Joo J, Kim M-J, Kim Y-K, Nahm BH, et al (2011) OsbHLH148, a basic helix-loop-helix, interacts with OsJAZ proteins in a jasmonate signaling pathway leading to drought tolerance in rice. Plant J 65: 907–921. 21332845 10.1111/j.1365-313X.2010.04477.x

[22] Yang D-L, Yao J, Mei C-S, Tong X-H, Zeng L-J, et al (2012) Plant hormone jasmonate prioritizes defense over growth by interfering with gibberellins signaling cascade. Proc Natl Acad Sci USA 109: E1192–E1200. 22529386 10.1073/pnas.1201616109 PMC3358897

[23] Ye M, Luo SM, Xie JX, Li YF, Xu T, et al (2012) Silencing COI1 in rice increases susceptibility to chewing insects and impairs inducible defense. PLoS ONE 7: e36214. 22558386 10.1371/journal.pone.0036214 PMC3338713

[24] Sheard LB, Tan X, Mao H, Wilters J, Ben-Nissan G, et al (2010) Jasmonate perception by inositol-phosphate-potentiated COI-JAZ co-receptor. Nature 468: 400–407. 20927106 10.1038/nature09430 PMC2988090

[25] Arnold K, Bordoli L, Kopp J, Schwede T (2006) The SWISS-MODEL Workspace: A web-based environment for protein structure homology modeling. Bioinformatics. 22: 195–201. 10.1093/bioinformatics/bti770 16301204

[26] DeLano WI (2002) The PyMOL Molecular Graphics System, DeLano Scientific LLC, San Carlos, CA.

[27] Murray MG, Thompson WF (1980) Rapid isolation of high molecular weight plant DNA. Nucleic Acids Research 8: 4321–4325. 7433111 10.1093/nar/8.19.4321 PMC324241

[28] Carpenter CD, Simon AE (1998) Preparation of RNA. Methods Mol Biol 82: 85–89. 9664416 10.1385/0-89603-391-0:85

