# Supplementary material for: Oryza sativa COI Homologues Restore Jasmonate Signal Transduction in Arabidopsis coi1-1 Mutants
Source: PLoS One. 2013 Jan 8;8(1):e52802. doi: 10.1371/journal.pone.0052802 (PMC3540053; doi:10.1371/journal.pone.0052802)
Supplement: Figure S1 — Molecular modeling of OsCOI-coronatine complex and OsCOI-JAZ interaction. (PDF) [file pone.0052802.s001.pdf]

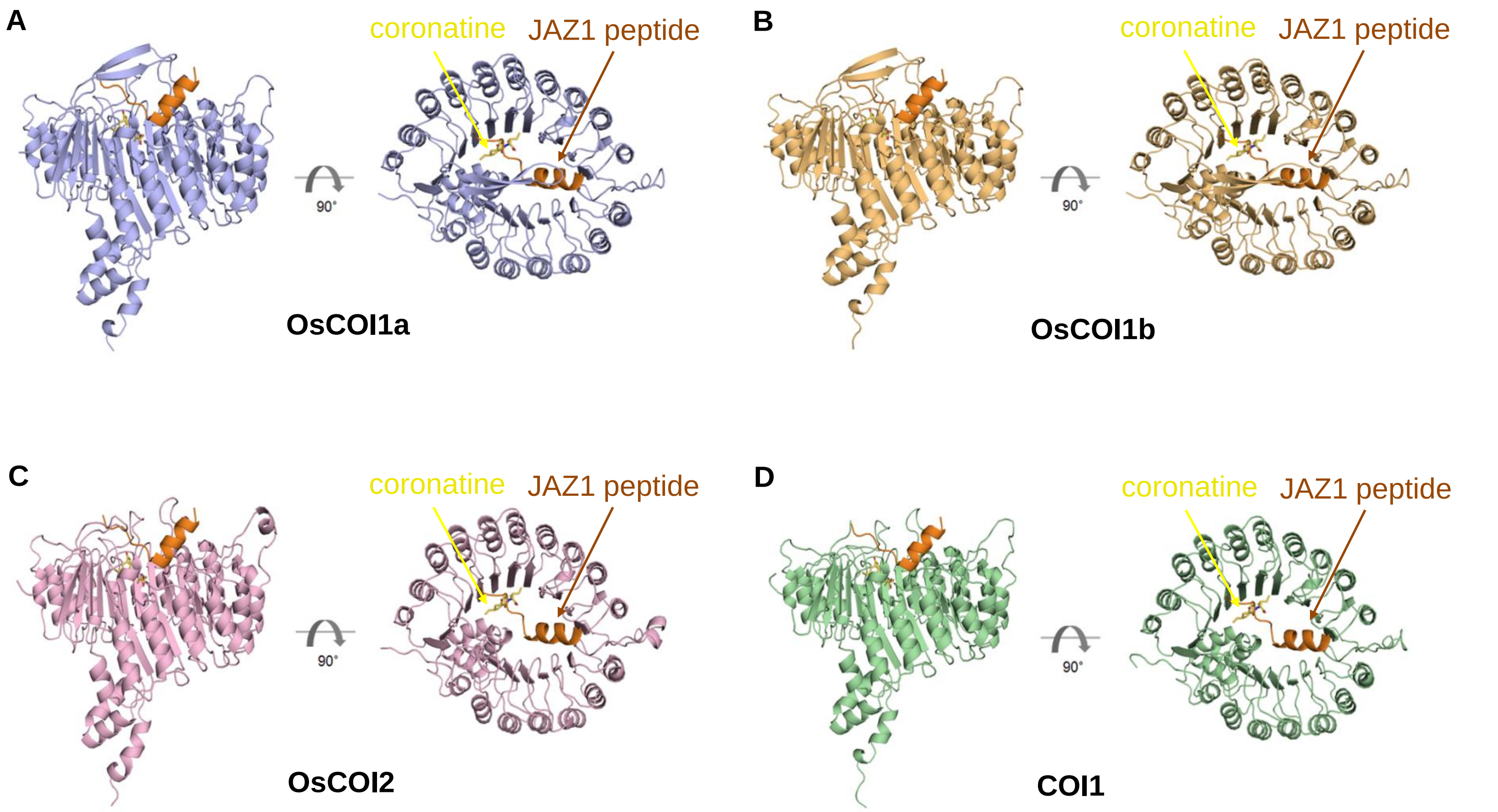

Figure S1. Molecular modeling of OsCOI-coronatine complex and OsCOI-JAZ interaction. Structure of OsCOI1a (blue in A), OsCOI1b (light-orange in B), OsCOI2 (pink in C), and COI1 (green in D) are shown by side and top views. Structure of complex was determined on the basis of COI1-JAZ1 complex of [24]. Coronatine (yellow) and JAZ1 peptide (reddish brown) binding to OsCOIs are shown.
