# Supplementary material for: Oryza sativa COI Homologues Restore Jasmonate Signal Transduction in Arabidopsis coi1-1 Mutants
Source: PLoS One. 2013 Jan 8;8(1):e52802. doi: 10.1371/journal.pone.0052802 (PMC3540053; doi:10.1371/journal.pone.0052802)
Supplement: Figure S2 — Molecular modeling of COI-coronatine complex. (PDF) [file pone.0052802.s002.pdf]

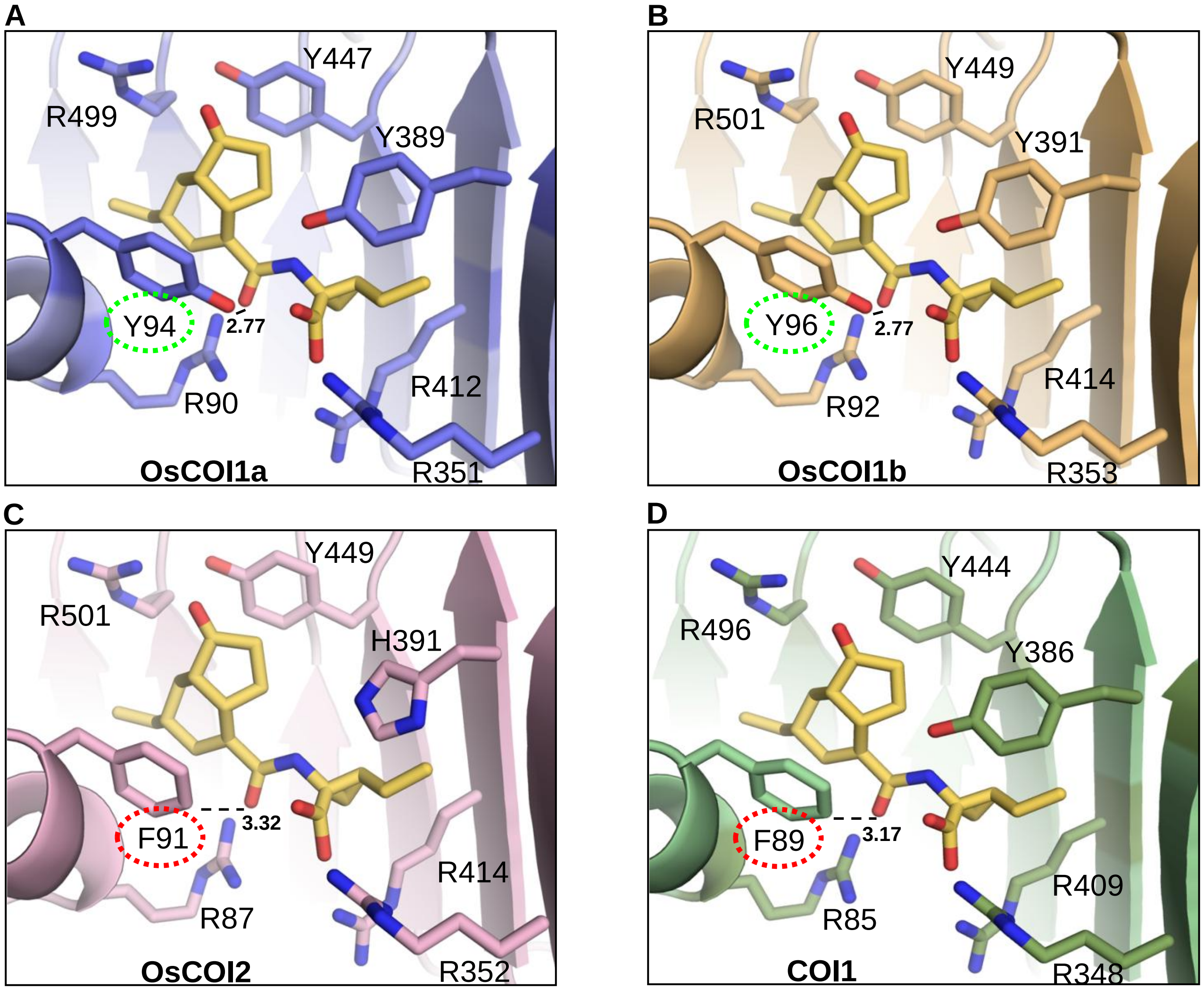

Figure S2. Molecular modeling of COI-coronatine complex. Complexes between coronatine (yellow) and OsCOI1a (blue in A), OsCOI1b (light orange in B), OsCOI2 (pink in C), COI1 (green in D), respectively, are shown. COI1 and OsCOI2 have Phe 89 or Phe91 residue but OsCOI1a and OsCOI1b have Tyr94 or Tyr96 residue, respectively. Hydrogen bonds are shown with dotted lines. COI1 and OsCOI2 form 3.17 Å and 3.32 Å hydrogen bond, respectively, but both OsCOI1a and OsCOI1b form 2.77 Å hydrogen bond.
