# Supplementary material for: Oryza sativa COI Homologues Restore Jasmonate Signal Transduction in Arabidopsis coi1-1 Mutants
Source: PLoS One. 2013 Jan 8;8(1):e52802. doi: 10.1371/journal.pone.0052802 (PMC3540053; doi:10.1371/journal.pone.0052802)
Supplement: Figure S3 — Molecular modeling of COI-JAZ interaction. (PDF) [file pone.0052802.s003.pdf]

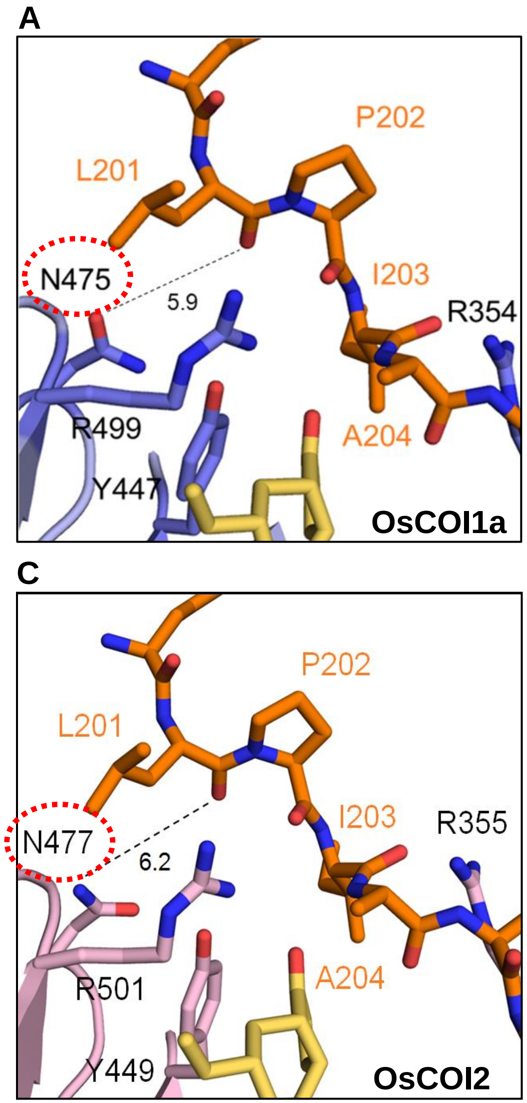

Figure S3. Molecular modeling of COI-JAZ interaction. Interaction between JAZ1 (yellow) and OsCOI1a (blue in A), OsCOI1b (light orange in B), OsCOI2 (pink in C) and COI1 (green in D) are shown. COI1 has Tyr472 residue, but OsCOIs have Asn475 or Asn477 residue at the site. Hydrogen bonds are shown with dotted lines. COI1 forms 3.1 Å hydrogen bond but OsCOIs form about 6 Å bonds. OsJAZ1 would form the same structure to OsCOI1-JAZ1 as in A-C.
