# Supplementary material for: Oryza sativa COI Homologues Restore Jasmonate Signal Transduction in Arabidopsis coi1-1 Mutants
Source: PLoS One. 2013 Jan 8;8(1):e52802. doi: 10.1371/journal.pone.0052802 (PMC3540053; doi:10.1371/journal.pone.0052802)
Supplement: Figure S4 — OsJAZs interact with OsCOIs in a coronatine-dependent manner in Y2H assays. (PDF) [file pone.0052802.s004.pdf]

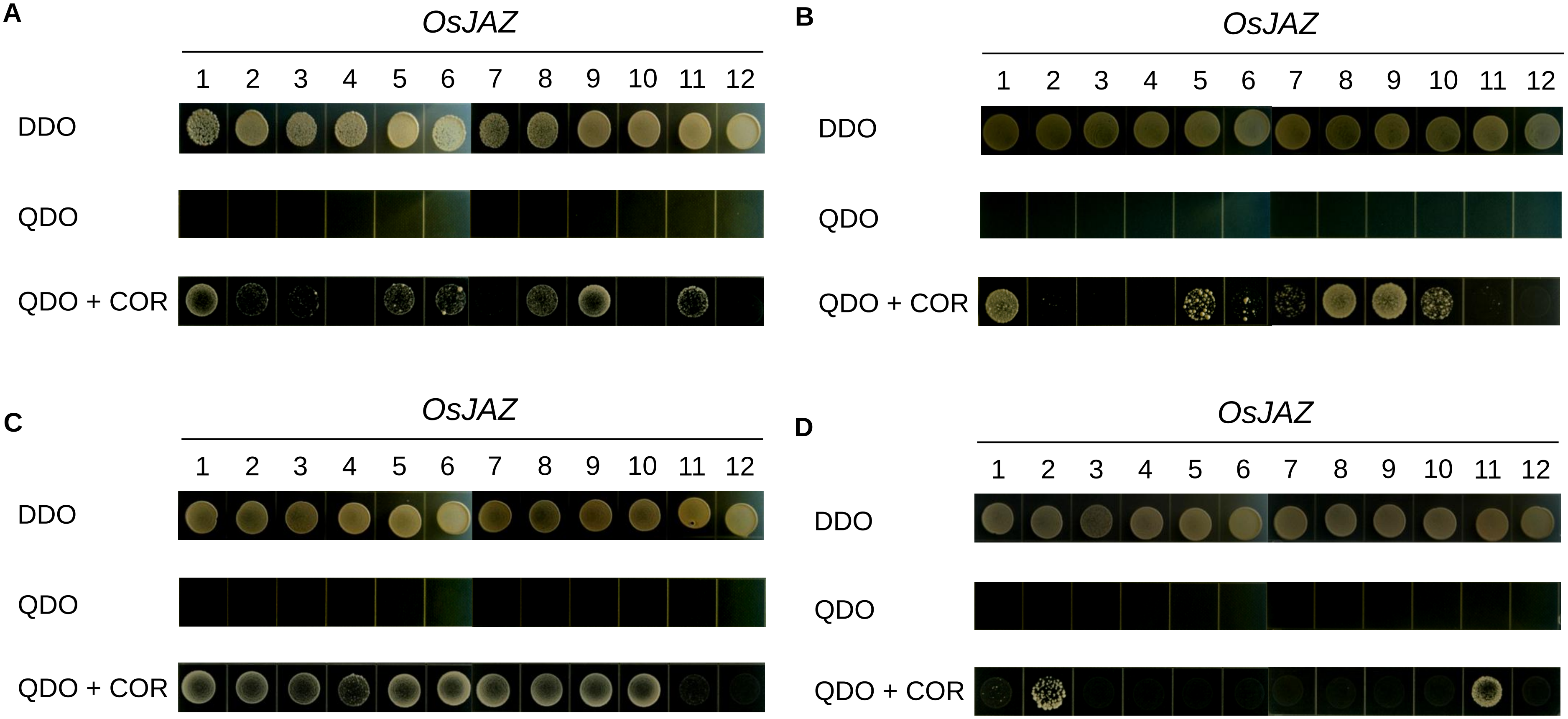

A
B
C
D
OsJAZ
1 2 3 4 5 6 7 8 9 10 11 12
DDO
QDO
QDO + COR

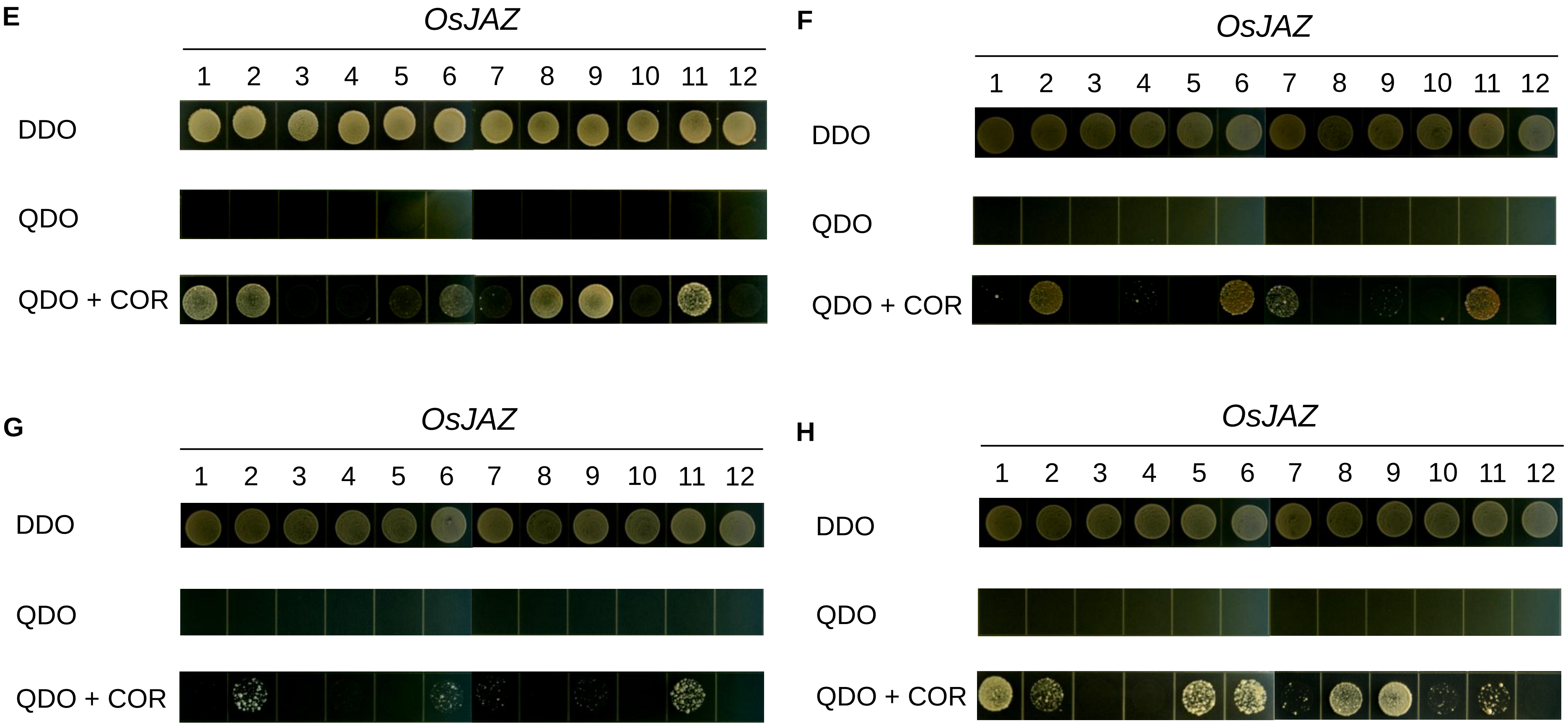

Figure S4. OsJAZs interact with OsCOIs in a coronatine-dependent manner in Y2H assay.
A, OsCOI1a-OsJAZs. B, OsCOI1a(N475Y)-OsJAZs. C, OsCOI1b-OsJAZs. D, OsCOI2-OsJAZs. E, OsCOI2(H391Y)-OsJAZs. F, OsCOI2(F91Y)-OsJAZs. G, OsCOI2(N477Y)-OsJAZs. H, OsCOI2(F91Y, H391Y, N477Y)-OsJAZs. *S. cerevisiae* AH109 carrying pGADT7-OsJAZ constructs (prey) and pGBKT7-OsCOIs (bait) was dropped on synthetic dropout glucose medium (SD) without Leu and Trp (DDO) as a co-transformation control. SD medium without Ade, His, Leu and Trp (QDO) was used to test protein interactions in the presence of coronatine (COR) at 100 μM. OsCOI1a(N475Y) is a point mutant in which asparagine at 475 has been changed to tyrosine. OsCOI2(H391Y) is a point mutant in which histidine at 391 has been changed to tyrosine. OsCOI2 (N477Y) is a point mutant in which asparagine at 477 has been changed to tyrosine. OsCOI2(F91Y) is a point mutant in which phenylalanine at 91 has been changed to tyrosine. OsCOI2(F91Y, H391Y, N477Y) is a point mutant in which each amino acid at there position has been changed to tyrosine.
