# Supplementary material for: Oryza sativa COI Homologues Restore Jasmonate Signal Transduction in Arabidopsis coi1-1 Mutants
Source: PLoS One. 2013 Jan 8;8(1):e52802. doi: 10.1371/journal.pone.0052802 (PMC3540053; doi:10.1371/journal.pone.0052802)
Supplement: Figure S6 — Expression of transgenes and restoration of JA response by complementation. (PDF) [file pone.0052802.s006.pdf]

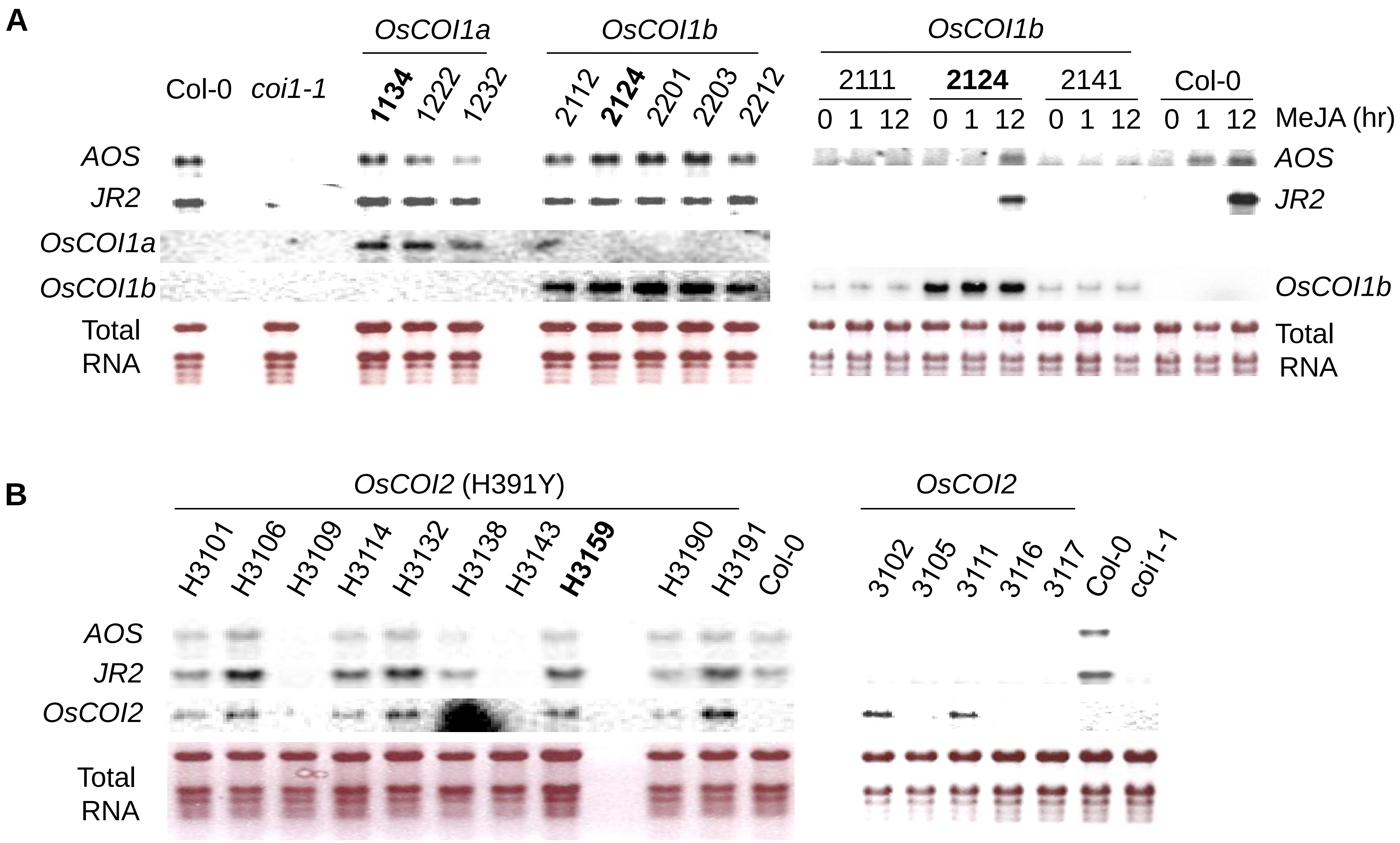

Figure S6. Expression of transgenes and restoration of JA response by complementation. A, Inducible expression of *AOS* and *JR2* by MeJA in *coi1-1* mutant transformed with *OsCOI1a* or *OsCOI1b* at T2 generation. B, Inducible expression of *AOS* or *JR2* by MeJA in *coi1-1* mutant transformed with *OsCOI2*(H391Y) or *OcCOI2* at T1 generation. Col-0 and *coi1-1 was also* treated with 50 µM MeJA. Total RNA was isolated at 6 hours after MeJA treatment unless specified. rRNA was visualized by ethidium bromide staining to show equal loading. Lines selected for further study was shown in a bold.
