# Supplementary material for: Oryza sativa COI Homologues Restore Jasmonate Signal Transduction in Arabidopsis coi1-1 Mutants
Source: PLoS One. 2013 Jan 8;8(1):e52802. doi: 10.1371/journal.pone.0052802 (PMC3540053; doi:10.1371/journal.pone.0052802)
Supplement: Figure S7 — Genomic Southern blot analysis of complemented coi1-1 mutants. (PDF) [file pone.0052802.s007.pdf]

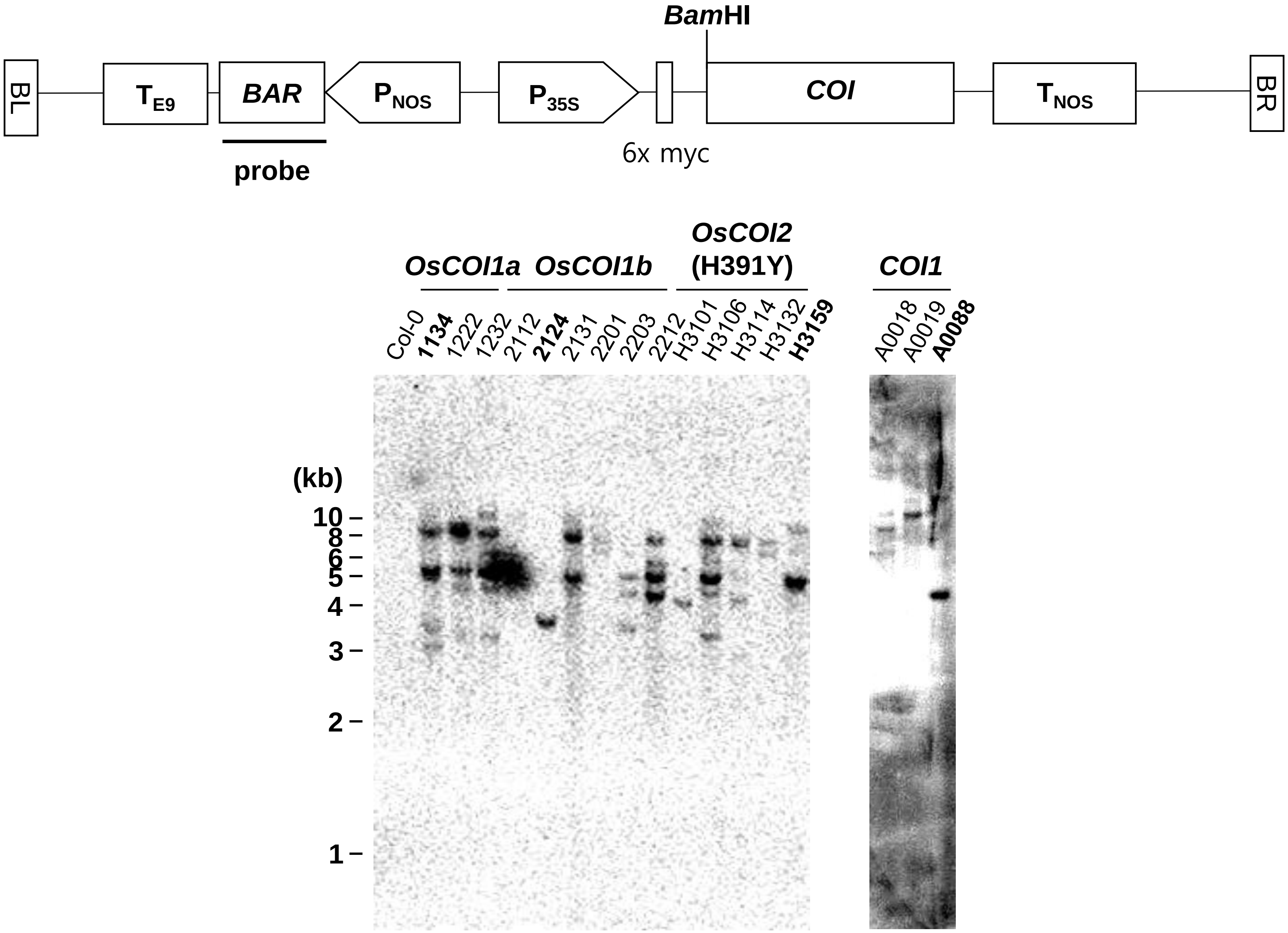

Figure S7. Genomic Southern blot analysis of complemented *coi1-1* mutants. In the recombinant *35S::COI* construct, coding region of the *COIs* were placed under the control of the *35S* promoter for constitutive overexpression, respectively. Genomic DNA was digested with *Bam*HI and the blot was hybridized with a *bar* probe. Transformant lines containing a single cop y of transgene and maintaining relatively high level of expression was selected (in bolds) for further studies.
