# Supplementary material for: Oryza sativa COI Homologues Restore Jasmonate Signal Transduction in Arabidopsis coi1-1 Mutants
Source: PLoS One. 2013 Jan 8;8(1):e52802. doi: 10.1371/journal.pone.0052802 (PMC3540053; doi:10.1371/journal.pone.0052802)
Supplement: Table S1 — The amino acid and nucleotide sequence identity of COI1 and OsCOIs. (PDF) [file pone.0052802.s008.pdf]

Table S1. The amino acids and nucleotide sequence identity of COI1 and OsCOIs.

A

Amino Acid		Percent Identity[1]			
		COI1	OsCOI1a	OsCOI1b	OsCOI2
Divergence[2]	COI1		55.5	56.5	54.5
	OsCOI1a	64.9		81.7	62.7
	OsCOI1b	63.0	20.8		62.9
	OsCOI2	66.1	48.1	47.4	

B

Nucleotide		Percent Identity			
		COI1	OsCOI1a	OsCOI1b	OsCOI2
Divergence	COI1		57.8	58.5	56.4
	OsCOI1a	60.9		82.5	68.3
	OsCOI1b	59.1	20.6		67.6
	OsCOI2	63.7	41.7	42.0	

[1]Percent Identity is direct sequence comparison by MeqAlign program in DNAstar software.
[2]Divergence was calculated by comparing sequence pairs in relation to the phylogeny reconstructed by MegAlign program in DNAstar software.
