# Supplementary material for: Oryza sativa COI Homologues Restore Jasmonate Signal Transduction in Arabidopsis coi1-1 Mutants
Source: PLoS One. 2013 Jan 8;8(1):e52802. doi: 10.1371/journal.pone.0052802 (PMC3540053; doi:10.1371/journal.pone.0052802)
Supplement: Table S2 — Summary of the Y2H assays with JAZs. (PDF) [file pone.0052802.s009.pdf]

Table S2. Summary of the Y2H assay of JAZ

	1	2	3	4	5	6	7	8	9	10	11	12
OsCOI1a	-[1]	-	++	++	-	-	-	-	+++	-	-	-
OsCOI1a(N475Y)[2]	+	-	++	++	-	-	-	-	+++	-	-	+
OsCOI1b	+++	+++	+++	+++	+	++	-	+	+++	-	+++	+++
OsCOI2	-	-	-	-	-	-	-	-	-	-	-	-
OsCOI2(H391Y)[3]	+++	+++	++	+++	-	-	-	-	+++	-	-	-
OsCOI2(F91Y)[4]	-	-	++	+	-	-	-	-	++	-	-	-
OsCOI2(N477Y)[5]	-	+	-	-	-	-	-	-	-	-	-	-
OsCOI2(F91Y, H391Y, N477Y)[6]	++	+++	+++	+++	-	-	-	-	+++	-	-	-
COI1[7]	+++	nd[8]	+++	nd	nd	nd	nd	nd	+++	++	nd	nd

[1]The strength of each interaction was rated as strong (+++), medium (++), weak (+) or undetectable (-), as shown in Figure S5.
[2]OsCOI1a(N475Y) is a point mutant in which asparagine at 475 has been changed to tyrosine.
[3]OsCOI2(H391Y) is a point mutant in which histidine at 391 has been changed to tyrosine.
[4]OsCOI2(F91Y) is a point mutant in which phenylalanine at 91 has been changed to tyrosine.
[5]OsCOI2(N477Y) is a point mutant in which asparagine at 477 has been changed to tyrosine.
[6]OsCOI2(F91Y, H391Y, N477Y) is a point mutant in which each amino acid at there position has been changed to tyrosine.
[7]Melato et al., 2008; Chung and Howe, 2009; Fonseca et al., 2009
[8]nd, not determined
